# Ionic characteristics and osmolarity of cooling fluid in hepatic laser ablation: a 7T MRI-based comparison in ex vivo bovine liver

**DOI:** 10.1007/s10103-026-05031-y

**Published:** 2026-09-11

**Authors:** Fiona Mankertz, Josephine Berger, Nour Maalouf, Benedikt Stenzl, Antonia Ashkar, Ole Gemeinhardt, Johannes Uhlig, Saif Afat, Norbert Hosten, Judith Herrmann

**Affiliations:** 1 Department of Diagnostic and Interventional Radiology, Universitätsklinikum Tübingen, Tübingen, Germany; 2 Department of General and Visceral Surgery, Charité - University Medicine Berlin, Berlin, Germany; 3 Institute for Diagnostic Radiology and Neuroradiology, Greifswald University Hospital, Greifswald, Germany

**Keywords:** Hepatic ablation, Laser ablation, Cooling fluid, Ablation volume, Ex vivo bovine liver model, MRI volumetry

## Abstract

To compare MRI-defined ablation volume among three cooling fluids with different ionic characteristics and osmolarity in ex vivo bovine liver tissue. In this ex vivo pilot study, 36 laser ablations were performed in five bovine livers using internal applicator cooling with 0.9% sodium chloride, 5% dextrose in water, or 10% mannitol at 60 mL/h. Post-ablation MRI was performed at 7 T. Primary endpoint was manual MRI volumetry; secondary endpoint was maximum axial diameter. Semi-automatic volumetry and intra-rater repeatability were assessed as supplementary analyses. Linear mixed-effects models with source liver as a random intercept were used for group comparisons. Overall, 33/36 ablations were evaluable. Mean ablation volume was 21.4 ± 1.5 cm³ with sodium chloride, 21.7 ± 1.4 cm³ with dextrose, and 23.6 ± 1.3 cm³ with mannitol. Cooling fluid group was associated with ablation volume (p = 0.002). Ablation volume was larger with mannitol than with sodium chloride (mean difference, 2.2 cm³; adjusted p < 0.001) or 5% dextrose (mean difference, 1.9 cm³; adjusted p = 0.006). Maximum axial diameter showed the same group pattern. Agreement between manual and semi-automatic volumetry was high (ICC, 0.96); intra-rater repeatability was excellent for volume (ICC, 0.98) and diameter (ICC, 0.97). In ex vivo bovine liver, 10% mannitol was associated with larger MRI-defined laser ablation volumes than 0.9% sodium chloride or 5% dextrose.

## Introduction

Hepatic thermal ablation is an established liver-directed treatment modality for selected patients with primary and secondary liver malignancies, particularly those who are poor candidates for surgical resection [1, 2]. Despite its clinical relevance for local tumor control, factors affecting ablation volume remain only partially characterized, and final ablation volume is not determined by nominal power and treatment time alone [3–6]. Once temperatures at the applicator-tissue interface exceed 100 °C, tissue vaporization and carbonization occur: this reduces energy transmission into surrounding parenchyma, and ablation volume growth becomes self-limiting [7, 8]. Internal applicator cooling was introduced to mitigate this effect, and experimental studies have shown that cooling reduces carbonization and permits larger coagulation volumes than uncooled systems [9–11]. Ablation geometry therefore depends not only on prescribed energy delivery but also on the local thermal conditions immediately surrounding the cooled applicator. In this setting, the cooling fluid is part of the thermal system and may be associated with the final extent of ablation.

MRI permits morphologic and volumetric assessment of ablation volume and is technically well suited to fibre-based laser systems because these applicators generate comparatively little susceptibility-related interference [8, 10, 12–15]. Prior work in hepatic laser ablation [LA] has focused predominantly on power settings, applicator geometry, cooling sheaths, and image guidance [16–18]. By comparison, evidence on the effect of cooling medium on ablation results is scarce. A previous ex vivo bovine study from the authors showed an effect of coolant composition on postprocedural ablation volume and carbonization. The fluids examined in that study were isotonic saline, distilled water, and a 50:50 mixture [19].

Ionic composition and osmolarity describe related but distinct solution characteristics. Ionic composition reflects the presence and concentration of charged species, whereas osmolarity reflects the total concentration of solute particles irrespective of their ionic or non-ionic nature [20]. Prior data therefore suggest that coolant composition is associated with ablation volume, but direct comparison of clinically used cooling fluids differing in ionic characteristics and osmolarity is lacking. The hypothesis was that 0.9% sodium chloride, 5% dextrose in water, and 10% mannitol would yield different ablation volumes. The aim of this study was to compare MRI-defined ablation volume among three cooling fluids with different ionic characteristics and osmolarity in ex vivo bovine liver tissue.

## Materials and methods

### Study design

This ex vivo comparative pilot study compared LA volume among cooling fluids differing in ionic characteristics and osmolarity in bovine liver tissue. The study compared three prespecified cooling fluid groups: 0.9% sodium chloride, 5% dextrose in water, and 10% mannitol. The primary endpoint was postprocedural ablation volume on MRI by manual volumetry. The secondary endpoint was maximum axial ablation diameter. Semi-automatic volumetry and intra-rater repeatability of manual volumetry and maximum axial ablation diameter were assessed as supplementary validation analyses.

Bovine livers were obtained from a commercial food-chain source [blinded for review] within 3 h of culling and processed on the day of acquisition. Reporting was informed by the ARRIVE 2.0 guidelines where applicable to the ex vivo design.

### Specimen procurement and preparation

Each specimen underwent macroscopic inspection by a certified veterinarian before inclusion. Adherent extrahepatic tissue and loose connective tissue were removed. Areas with capsular disruption, focal contusion, major parenchymal tears, or marked heterogeneity were excluded.

All ablations were performed in intact whole-liver specimens. Before intervention, livers were kept in a heated saline bath at 38 °C to approximate in vivo thermal conditions. Local parenchymal temperature was measured with a probe thermometer inserted to a depth of approximately 10 mm adjacent to the planned applicator path. Procedures were performed only when parenchymal temperature was between 37° and 39 °C. If multiple ablations were performed within the same liver, all sites were marked in advance, with a center-to-center distance of at least 100 mm. For each ablation, the liver from which it originated and the site position were recorded.

The cooling fluids, 0.9% sodium chloride, 5% dextrose in water, and 10% mannitol, were used without dilution or admixture. 10% mannitol was selected as a commercially available non-ionic infusion solution with a substantially higher nominal osmolarity than the other study fluids, thereby broadening the physicochemical range evaluated while allowing use of an undiluted clinically available formulation. Ablations were assigned according to a prespecified repeating 1:1:1 allocation sequence across the three cooling fluids.

### Ablation protocol

LA was performed using a Medilas Fibertom 5100 system (Dornier MedTech Europe GmbH, Munich, Germany) in conjunction with a standard RoweCath applicator (RoweMed, Parchim, Germany). Applicator placement was performed manually without image guidance. The fibre was advanced along the prespecified trajectory into the hepatic parenchyma to the planned treatment center. Internal applicator cooling represented the experimental variable. According to group allocation, the applicator was perfused with 0.9% sodium chloride, 5% dextrose in water, or 10% mannitol at a constant flow rate of 60 mL/h throughout ablation. Laser settings were constant across groups. Energy was delivered at 25 W for 10 min, corresponding to 15,000 J per ablation. Standardized ablation, MRI acquisition, and image analysis parameters are summarized in Table 1. After treatment, the fibre and applicator were withdrawn along the insertion path.

**Table 1:** Technical ablation, MRI acquisition, and image analysis parameters. Ablation and imaging parameters were standardized across all experimental groups except for the internal applicator cooling fluid. In-plane resolution and voxel size were derived from the field of view, acquisition matrix, and slice thickness. Abbreviations: FL2D, two-dimensional fast low-angle shot; FL3D, three-dimensional fast low-angle shot; MRI, magnetic resonance imaging; Nd:YAG, neodymium-doped yttrium aluminium garnet; ROI, region of interest; SD, standard deviation; T1w, T1-weighted; T2w, T2-weighted; TSE, turbo spin echo; VIBE, volumetric interpolated breath-hold examination

Parameters	Values
Ablation parameters	
Laser system	Medilas Fibertom 5100 Nd: YAG (Dornier MedTech Europe GmbH, Munich, Germany)
Laser wavelength	1064 nm
Optical fibre and diffuser tip	Dornier Diffuser Tip H6111-T3 (Dornier MedTech Europe GmbH, Munich, Germany)
Diffusion catheter outer diameter	5.5 F, 1.8 mm
Applicator system	RoweCath (RoweMed AG, Parchim, Germany)
Laser power	25 W
Treatment duration	10 min
Delivered energy	15,000 J
Internal applicator cooling flow rate	60 mL/h
Measured parenchymal temperature before ablation, mean ± SD (range)	38.1 ± 0.4 (37.3–38.8) °C
Minimum center-to-center distance between ablation sites	100 mm
Planned time from ablation to MRI	10 min
MRI acquisition parameters	
MRI system	7T BioSpec 70/30 (Bruker BioSpin GmbH, Ettlingen, Germany)
MRI slider	Bio R.Bed 72 MRI Slider (Bruker BioSpin GmbH, Ettlingen, Germany)
MRI coil	¹H receive-only 8 × 1 mouse body surface array coil
Field of view	80 × 80 mm
Acquisition matrix	256 × 256
In-plane resolution	0.31 × 0.31 mm
Slice thickness	1.5 mm
Interslice gap	0 mm
Voxel size	0.31 × 0.31 × 1.5 mm³
MRI sequences and orientations	T1w FL2D: axial, coronal, sagittal T1w FL3D VIBE: axial T2w TSE: axial, coronal
Image analysis parameters	
Manual volumetry	Closed-polygon ROI segmentation on each evaluable slice
Semi-automatic volumetry	Closed-polygon ROI interpolation using “Generate Missing ROIs” function
Volume calculation	Σ ROI area × 1.5 mm
Diameter	Maximum in-plane ablation-zone diameter on axial T1-weighted FL2D
Software	Horos DICOM Viewer v3.3.5 (Horos Project, Annapolis, MD, USA)

### Image acquisition

After ablation, the treated region was excised en bloc with an approximately 50-mm rim of surrounding non-ablated tissue. Each specimen was wrapped in cellophane and mounted on a Bruker BioSpin MRI slider (Bio R.Bed 72 MRI Slider) in fixed longitudinal orientation. MRI acquisition was set to approximately 10 min after completion of ablation. The exact interval was recorded for each specimen.

MRI was performed on a 7T BioSpec 70/30 system (Bruker BioSpin GmbH, Ettlingen, Germany) using a ¹H receive-only 8 × 1 mouse body surface array coil. The post-ablation protocol comprised T1-weighted flash 2-dimensional (FL2D) sequences in axial, coronal, and sagittal orientation, T1-weighted FL3D VIBE in axial orientation, and T2-weighted TSE sequences in axial and coronal orientation. For all sequences, field of view was 80 × 80 mm, acquisition matrix 256 × 256, slice thickness 1.5 mm, and interslice gap 0 mm. Image datasets were exported, assigned study codes, and reviewed in randomized order.

Axial T1-weighted FL2D images were used as the prespecified source series for volumetry because they provided the most consistent ablation-to-parenchyma contrast in the present model. Eligibility for volumetric analysis required a continuous, clearly marginated T1-hyperintense ablation zone on the prespecified axial T1-weighted FL2D source series. Ablations were excluded if image acquisition failed or if the ablation zone could not be reliably delineated. Eligibility was assessed independently by two readers with over 10 years of experience in MR image analysis, both blinded to cooling fluid group, with consensus in discrepant cases.

### Image analysis

Quantitative image analysis was performed by the primary reader using Horos DICOM Viewer v3.3.5 (Horos Project, Annapolis, MD, USA; RRID: SCR_017340). Quantitative volumetry and diameter measurements were performed by a single reader; inter-reader reproducibility was not part of the prespecified analysis. Manual volumetry was predefined as the primary measurement method. The ablation volume was contoured slice by slice on axial T1-weighted FL2D images using the closed polygon tool. The segmented area on each slice was multiplied by slice thickness, and slice volumes were summed to obtain total ablation volume in cm³. Maximum axial ablation diameter was recorded on the slice showing the greatest ablation extent. Semi-automatic volumetry was performed as a supplementary analysis using the “Generate Missing ROIs” function in Horos. For assessment of intra-rater repeatability, the primary reader repeated manual volumetry and maximum axial diameter measurements after a 4-week washout period using the same datasets. Macroscopic carbonization was documented qualitatively during specimen assessment but was not prospectively graded using a standardized macroscopic or histological scoring system and was therefore not included as a quantitative endpoint.

### Statistical analysis

Statistical analyses were performed using R version 3.6.1 (R Foundation for Statistical Computing, Vienna, Austria; RRID: SCR_001905) and IBM SPSS Statistics for Windows version 26.0 (IBM Corp., Armonk, NY, USA; RRID: SCR_016479). Continuous variables were summarized using mean ± standard deviation and median with interquartile range to describe both average values and distributional characteristics in this small experimental dataset. Because multiple ablations could originate from the same liver specimen, the primary analysis used a linear mixed-effects model with cooling fluid group as fixed effect and source liver as a random intercept. Variance components for the liver-level random intercept and residual error were extracted from each model. Standardized effect sizes were calculated for the pairwise group comparisons based on the model-derived mean differences and residual standard deviation. Model assumptions were assessed by visual inspection of quantile-quantile plots of the residuals and residual-versus-fitted plots for approximate normality and homoscedasticity. Ablation volume was the dependent variable. Estimated marginal means with 95% confidence intervals were derived from the model, and prespecified pairwise group comparisons were adjusted using Tukey correction. Maximum axial ablation diameter was analyzed using an analogous mixed-effects model. Semi-automatic volumetry was analyzed descriptively and compared with manual volumetry by intraclass correlation coefficient (ICC) and Bland-Altman assessment. Intra-rater repeatability of manual volumetry and maximum axial ablation diameter was quantified using two-way mixed-effects ICCs for absolute agreement, single measures, with 95% confidence intervals. Inferential group comparisons were two-sided, with *p* < 0.05 considered statistically significant. ICCs were reported with 95% confidence intervals; ICC p values tested whether the ICC was greater than 0.

## Results

**Fig. 1:**
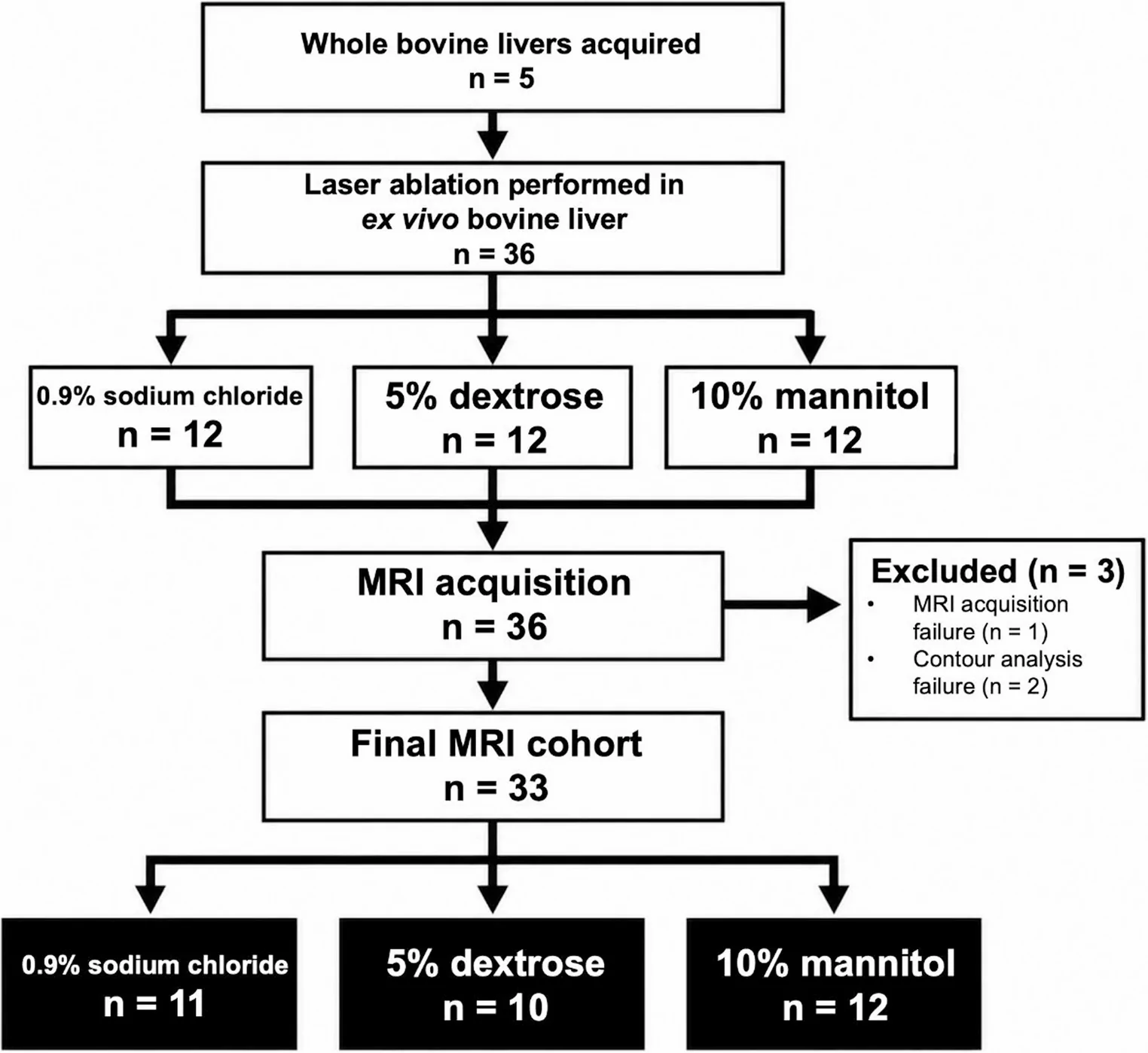
Study flow. Whole bovine livers obtained from a commercial food-chain source were used for 36 laser ablations in ex vivo bovine liver tissue. Ablations were allocated equally to internal applicator cooling with 0.9% sodium chloride (NaCl), 5% dextrose in water, or 10% mannitol. All ablations underwent postprocedural magnetic resonance imaging (MRI). Three ablations were excluded from MRI-based analysis because of MRI acquisition failure (*n* = 1) or failure of reliable contour analysis on the prespecified T1-weighted source series (*n* = 2). The final MRI cohort comprised 33 evaluable ablations

**Fig. 2:**
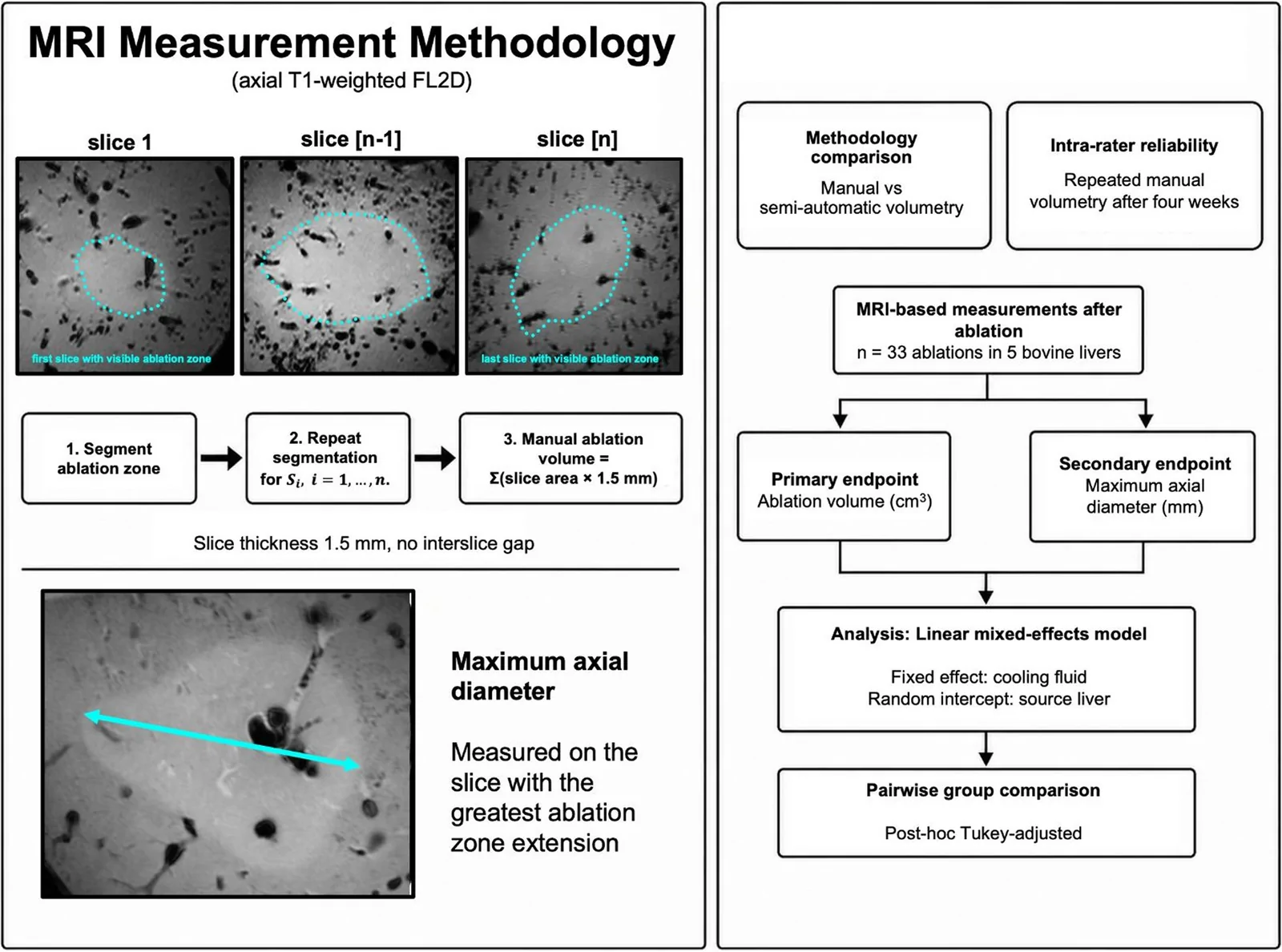
MRI measurement methodology and statistical analysis workflow. Post-ablation magnetic resonance imaging (MRI) was assessed on the axial T1-weighted FL2D source series. Manual ablation volume was determined by segmenting the visible ablation zone on each contiguous slice and summing the segmented slice areas multiplied by the slice thickness of 1.5 mm. Maximum axial diameter was measured on the slice showing the greatest ablation zone extension. Manual volumetry was compared with semi-automatic volumetry, and intra-rater reliability was assessed by repeated manual volumetry after a 4-week interval. The primary endpoint was MRI-based ablation volume in cm³. The secondary endpoint was maximum axial diameter in mm. Statistical analysis used a linear mixed-effects model with cooling fluid as fixed effect and source liver as random intercept, followed by Tukey-adjusted post hoc pairwise group comparisons

A total of 36 ablations were performed in 5 bovine livers and allocated equally across the three cooling fluid groups: 0.9% sodium chloride, 5% dextrose in water, and 10% mannitol. After application of the prespecified eligibility criteria for MRI-based volumetry, 33 ablations were included in the imaging endpoint analyses. Three ablations were excluded: two because the ablation zone could not be contoured reliably on the prespecified T1-weighted source series and one because of technical failure during image acquisition (Fig. 1). Exclusions occurred as follows: one in the 0.9% sodium chloride group and two in the 5% dextrose in water group. The final evaluable dataset comprised 11 ablations in the 0.9% sodium chloride group, 10 in the 5% dextrose in water group, and 12 in the 10% mannitol group. MRI-based measurement workflow is illustrated in Fig. 2.

Median number of ablations per liver was 7 (range, 6–8). Mean parenchymal temperature immediately before ablation was 38.1 °C (SD, 0.4) overall, with group-specific values of 38.1 °C (SD, 0.4) for 0.9% sodium chloride, 38.0 °C (SD, 0.4) for 5% dextrose in water, and 38.2 °C (SD, 0.3) for 10% mannitol. The mean interval from completion of ablation to the start of MRI acquisition was 10.4 min (SD, 1.2). Procedural characteristics and MRI-based outcome summaries are provided in Table 2. Macroscopic carbonization was observed in all three cooling-fluid groups. Because carbonization was not prospectively assessed using a standardized grading system, no quantitative between-group comparison was performed.

**Table 2:** Cooling-fluid characteristics. Cooling fluids were used as supplied, without dilution or admixture. Composition, electrolyte content, pH, and nominal osmolarity are reported according to manufacturer product information. Ionic status was assigned according to the presence or absence of dissociated electrolytes in solution. Nominal osmolarity is reported in mOsm/L and was not independently measured. Abbreviations: Cl⁻, chloride; Na⁺, sodium; mOsm/L, milliosmoles per liter

	0.9% sodium chloride	5% dextrose in water	10% mannitol
Product name	Isotone Kochsalz-Lösung 0,9% Braun Infusionslösung	Glucose 5% B. Braun Infusionslösung	Mannit-Lösung 10% Infusionslösung
Manufacturer	B. Braun Melsungen AG, Melsungen, Germany	B. Braun Melsungen AG, Melsungen, Germany	SERAG-WIESSNER GmbH & Co. KG, Naila, Germany
Concentration	Sodium chloride 9.0 g/L	Glucose 50 g/L	Mannitol 100 g/L
Ionic status	Ionic	Non-ionic	Non-ionic
Electrolyte content	Na⁺ 154 mmol/L; Cl⁻ 154 mmol/L	None	None
Nominal osmolarity	308 mOsm/L	278 mOsm/L	550 mOsm/L
pH	4.5-7.0	3.5–5.5	3.6–6.6

### Ablation volume and maximum axial diameter

**Fig. 3:**
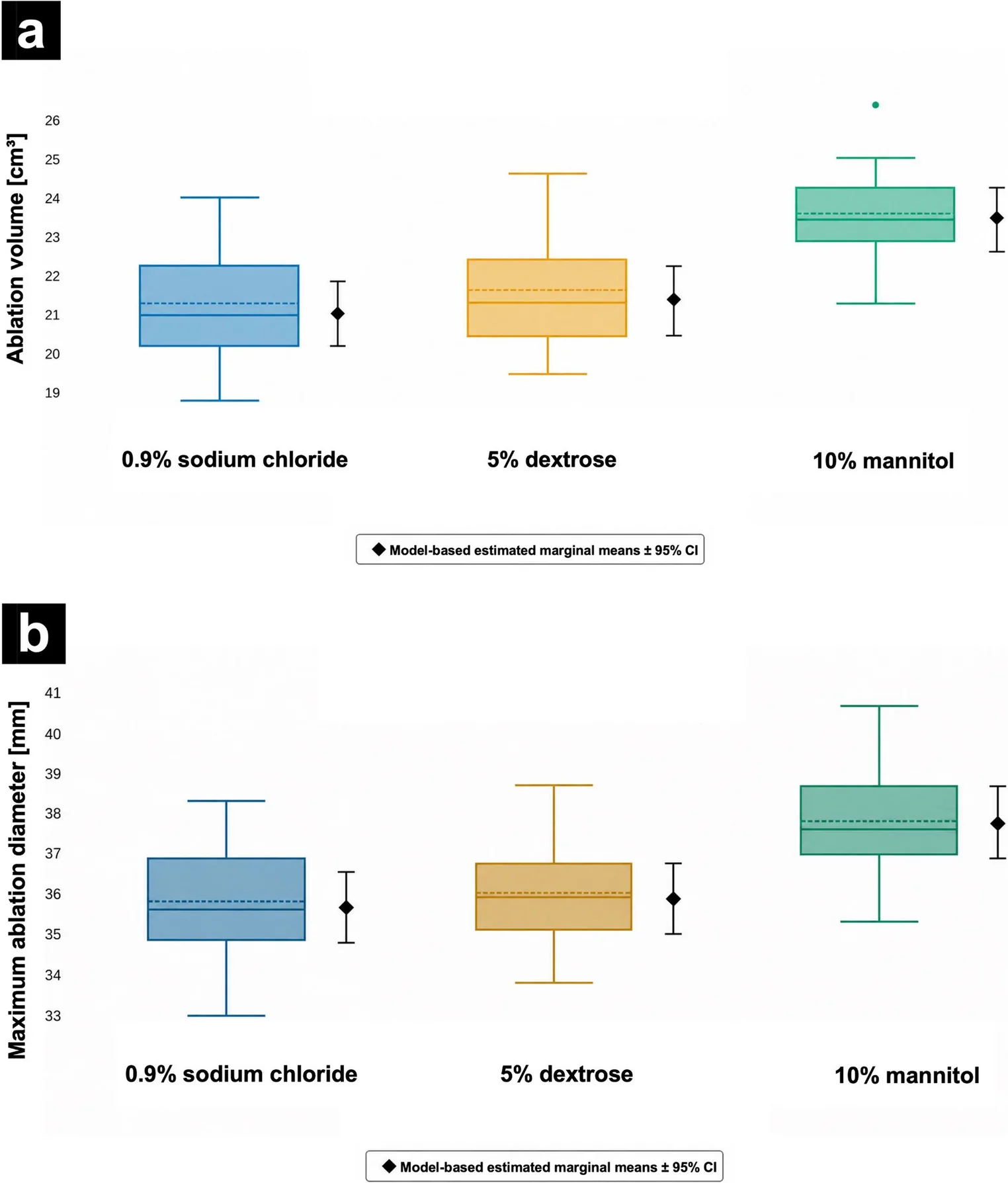
(**A**) MRI-defined ablation volume according to cooling fluid. Box-and-whisker plots show manual ablation volume for 0.9% sodium chloride (*n* = 11), 5% dextrose in water (*n* = 10), and 10% mannitol (*n* = 12). Boxes indicate interquartile range, solid lines medians, whiskers data range, and dashed lines group means. Black diamonds show model-based estimated marginal means with 95% confidence intervals. Mean ablation volume was 21.4 ± 1.5 cm³, 21.7 ± 1.4 cm³, and 23.6 ± 1.3 cm³, respectively. Cooling fluid group was associated with ablation volume (*p* = 0.002), with larger volumes for 10% mannitol than for 0.9% sodium chloride (adjusted *p* < 0.001) and 5% dextrose in water (adjusted *p* = 0.006). (**B**) Maximum axial ablation diameter according to cooling fluid. Box-and-whisker plots show maximum axial diameter for 0.9% sodium chloride (n = 11), 5% dextrose in water (n = 10), and 10% mannitol (n = 12). Boxes indicate interquartile range, solid lines medians, whiskers data range, and dashed lines group means. Black diamonds show model-based estimated marginal means with 95% confidence intervals. Mean maximum axial diameter was 35.8 ± 1.6 mm, 36.0 ± 1.5 mm, and 37.8 ± 1.4 mm, respectively. Cooling fluid group was associated with maximum axial diameter (p = 0.003), with larger diameters for 10% mannitol than for 0.9% sodium chloride (adjusted p = 0.007) and 5% dextrose in water (adjusted p = 0.016)

Among the 33 evaluable ablations, 11 were included in the 0.9% sodium chloride group, 10 in the 5% dextrose in water group, and 12 in the 10% mannitol group. Manual MRI volumetry of the ablation volume is summarized in Table 3; Fig. 3A. Mean ablation volume was 21.4 cm³ (SD, 1.5) in the 0.9% sodium chloride group, 21.7 cm³ (SD, 1.4) in the 5% dextrose in water group, and 23.6 cm³ (SD, 1.3) in the 10% mannitol group. Median values with interquartile ranges are provided in Table 3. In the primary linear mixed-effects model with source liver as a random intercept, cooling fluid group was associated with ablation volume (*p* = 0.002). The estimated liver-level random-intercept variance was 0.24 (cm^3^)^2^, and the residual variance was 1.91 (cm^3^)^2^. Model-based estimated marginal means were 21.4 cm³ (95% CI, 20.5–22.3) for 0.9% sodium chloride, 21.7 cm³ (95% CI, 20.8–22.6) for 5% dextrose in water, and 23.6 cm³ (95% CI, 22.7–24.5) for 10% mannitol. Prespecified pairwise comparisons with Tukey correction showed a mean difference of 2.2 cm³ (95% CI, 0.9–3.5; adjusted *p* < 0.001) between 10% mannitol and 0.9% sodium chloride, 1.9 cm³ (95% CI, 0.6–3.2; adjusted *p* = 0.006) between 10% mannitol and 5% dextrose in water, and − 0.3 cm³ (95% CI, -1.5 to 0.9; adjusted *p* = 0.81) between 0.9% sodium chloride and 5% dextrose in water.

**Table 3:** MRI-based outcomes and procedural characteristics by cooling fluid. Values are number, mean ± standard deviation, or median with interquartile range unless otherwise indicated. Estimated marginal means and p-values were derived from linear mixed-effects models with cooling fluid as fixed effect and source liver as random intercept. Ablations per liver are summarized within each column; therefore, group-specific medians do not sum to the overall median. ** *p* < 0.01. Abbreviations: CI = confidence interval; IQR = interquartile range; MRI = magnetic resonance imaging; SD = standard deviation

	Overall (*n* = 33)	0.9% sodium chloride (*n* = 11)	5% dextrose in water (*n* = 10)	10% mannitol (*n* = 12)
Evaluable ablations per liver, median (range), *n*	7 (6–8)	2 (2–3)	2 (2–3)	2 (2–3)
Pre-ablation temperature, mean ± SD, °C	38.1 ± 0.4	38.1 ± 0.4	38.0 ± 0.4	38.2 ± 0.3
Time from ablation to MRI, mean ± SD, min	10.4 ± 1.2	10.2 ± 1.1	10.7 ± 1.4	10.3 ± 1.1
Manual ablation volume, mean ± SD, cm³	22.3 ± 1.7	21.4 ± 1.5	21.7 ± 1.4	23.6 ± 1.3
Manual ablation volume, median (IQR), cm³	22.3 (20.9–23.5)	21.2 (20.4–22.3)	21.5 (20.7–22.4)	23.4 (22.8–24.3)
Estimated marginal mean (95% CI), cm³; global p-value	*p* = 0.002 **	21.4 (20.5–22.3)	21.7 (20.8–22.6)	23.6 (22.7–24.5)
Maximum axial diameter, mean ± SD, mm	36.6 ± 1.8	35.8 ± 1.6	36.0 ± 1.5	37.8 ± 1.4
Maximum axial diameter, median (IQR), mm	36.8 (35.3–37.7)	35.6 (34.7–36.9)	35.9 (35.0-36.8)	37.6 (36.9–38.6)
Estimated marginal mean (95% CI), mm; global p-value	*p* = 0.003 **	35.8 (34.9–36.7)	36.0 (35.1–36.9)	37.8 (36.9–38.7)

Mean maximum diameter was 35.8 mm (SD, 1.6) in the 0.9% sodium chloride group, 36.0 mm (SD, 1.5) in the 5% dextrose in water group, and 37.8 mm (SD, 1.4) in the 10% mannitol group (Table 2; Fig. 3B**).** Median maximum diameters were 35.6 mm (IQR, 34.7–36.9), 35.9 mm (IQR, 35.0-36.8), and 37.6 mm (IQR, 36.9–38.6), respectively. In the corresponding mixed-effects model, cooling fluid group was associated with maximum axial diameter (*p* = 0.003). The estimated liver-level random-intercept variance was 0.20 mm², and the residual variance was 2.10 mm². Model-based estimated marginal means were 35.8 mm (95% CI, 34.9–36.7) for 0.9% sodium chloride, 36.0 mm (95% CI, 35.1–36.9) for 5% dextrose in water, and 37.8 mm (95% CI, 36.9–38.7) for 10% mannitol. Post hoc pairwise comparisons showed larger ablation volume and maximum axial diameter with 10% mannitol than with both comparator fluids, whereas 0.9% sodium chloride and 5% dextrose in water did not differ significantly (Table 4). Residual diagnostics showed no substantial deviations from normality or homoscedasticity for either mixed-effects model.

**Table 4:** Tukey-adjusted post hoc pairwise comparisons. Pairwise comparisons are derived from linear mixed-effects models with cooling fluid as fixed effect and source liver as random intercept. Mean differences are reported in the unit of the corresponding outcome, cm³ for manual ablation volume and mm for maximum axial diameter. Positive values indicate higher measurements in the first-listed cooling-fluid group. Significance levels are indicated as *p < 0.05, **p < 0.01, and ***p < 0.001. Abbreviations: CI, confidence interval

Outcome	Comparison	Mean difference (95% CI)	Standardized effect size	Adjusted *p*-value
Manual ablation volume, cm³	10% mannitol vs. 0.9% sodium chloride	2.2 (0.9–3.5)	1.59	< 0.001 ***
	10% mannitol vs. 5% dextrose in water	1.9 (0.6–3.2)	1.37	0.006 **
	0.9% sodium chloride vs. 5% dextrose in water	-0.3 (-1.5–0.9)	-0.22	0.81
Maximum axial diameter, mm	10% mannitol vs. 0.9% sodium chloride	2.0 (0.6–3.4)	1.38	0.007 **
	10% mannitol vs. 5% dextrose in water	1.8 (0.4–3.2)	1.24	0.016 *
	0.9% sodium chloride vs. 5% dextrose in water	-0.2 (-1.5–1.1)	-0.14	0.93

### Validation and repeatability analyses

**Fig. 4:**
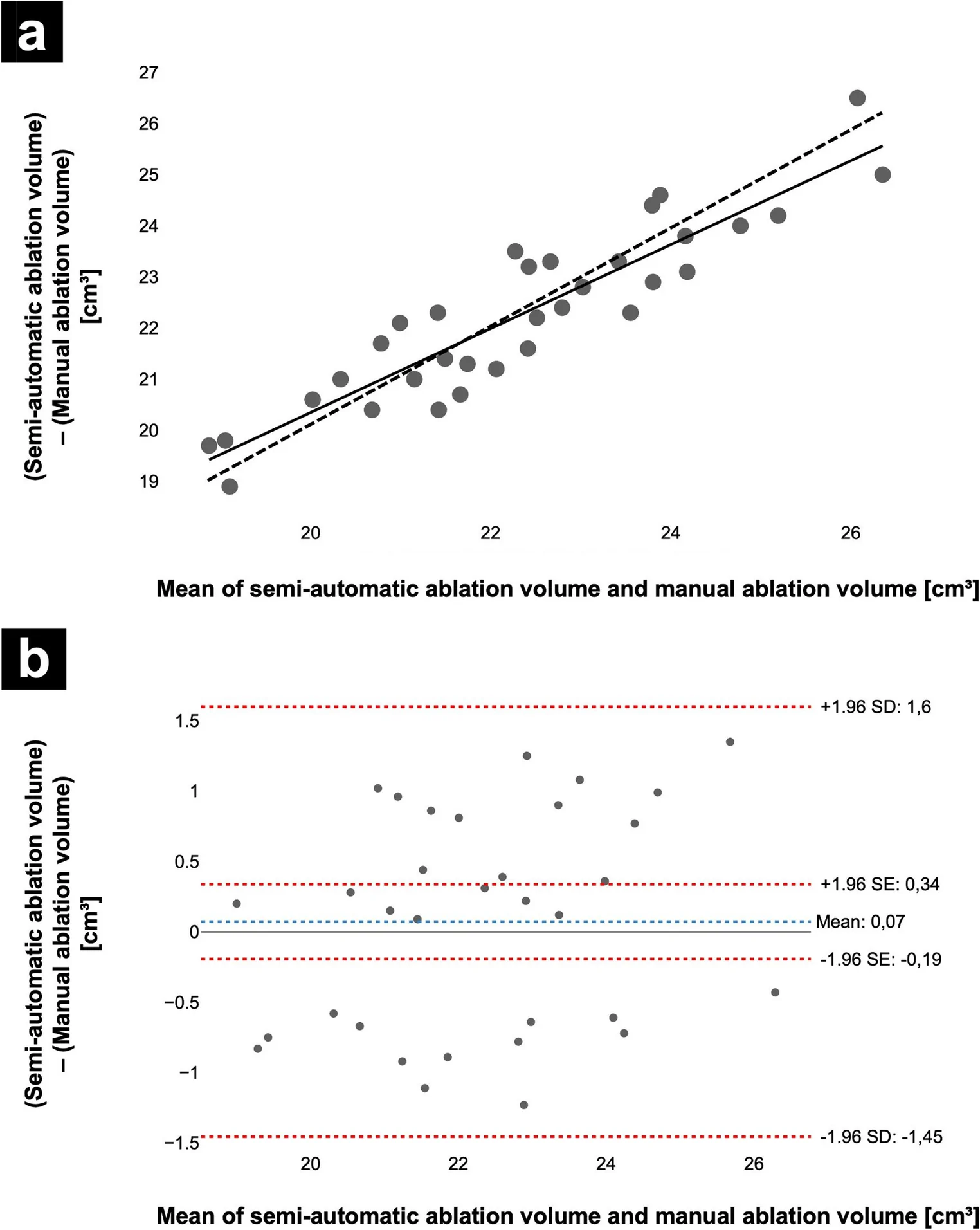
(**A**) Agreement between manual and semi-automatic MRI volumetry. Scatter plot comparing manual and semi-automatic ablation volume measurements in 33 evaluable ablations. The dashed line indicates the identity line, and the solid line indicates the fitted regression line. Agreement between methods was high, with an intraclass correlation coefficient of 0.96 (95% CI, 0.92–0.98). The mean absolute difference between methods was 0.19 ± 0.13 cm³. (**B**) Bland-Altman analysis of manual and semi-automatic MRI volumetry. Differences between semi-automatic and manual ablation volume measurements are plotted against the mean of both methods for 33 evaluable ablations. The blue dashed line indicates mean bias (0.07 cm³), and the red dashed lines indicate the 95% limits of agreement (-1.45 to 1.60 cm³), calculated as mean difference ± 1.96 standard deviations

Semi-automatic volumetry was available in all 33 evaluable ablations. Mean semi-automatic ablation volume was 21.6 cm³ (SD, 1.5) in the 0.9% sodium chloride group, 21.9 cm³ (SD, 1.4) in the 5% dextrose in water group, and 23.8 cm³ (SD, 1.4) in the 10% mannitol group. Agreement between manual and semi-automatic volumetry is shown in Fig. 4. Across all evaluable ablations, agreement between manual and semi-automatic volumetry was excellent, with an ICC of 0.96 (95% CI, 0.92–0.98; *p* < 0.001 for ICC > 0). The mean absolute difference between methods was 0.19 cm³ (SD, 0.13). Bland-Altman analysis showed a mean bias of 0.07 cm³, with 95% limits of agreement from − 1.45 to 1.60 cm³.

Repeated manual volumetry and repeated maximum axial diameter measurements were available in all 33 evaluable ablations after the 4-week washout period. The ICC for repeated manual volumetry was 0.98 (95% CI, 0.96–0.99), with a mean absolute difference of 0.15 cm³ (SD, 0.10) and a mean relative difference of 0.8%. For maximum axial diameter, the ICC for repeated measurements was 0.97 (95% CI, 0.94–0.99).

## Discussion

**Fig. 5:**
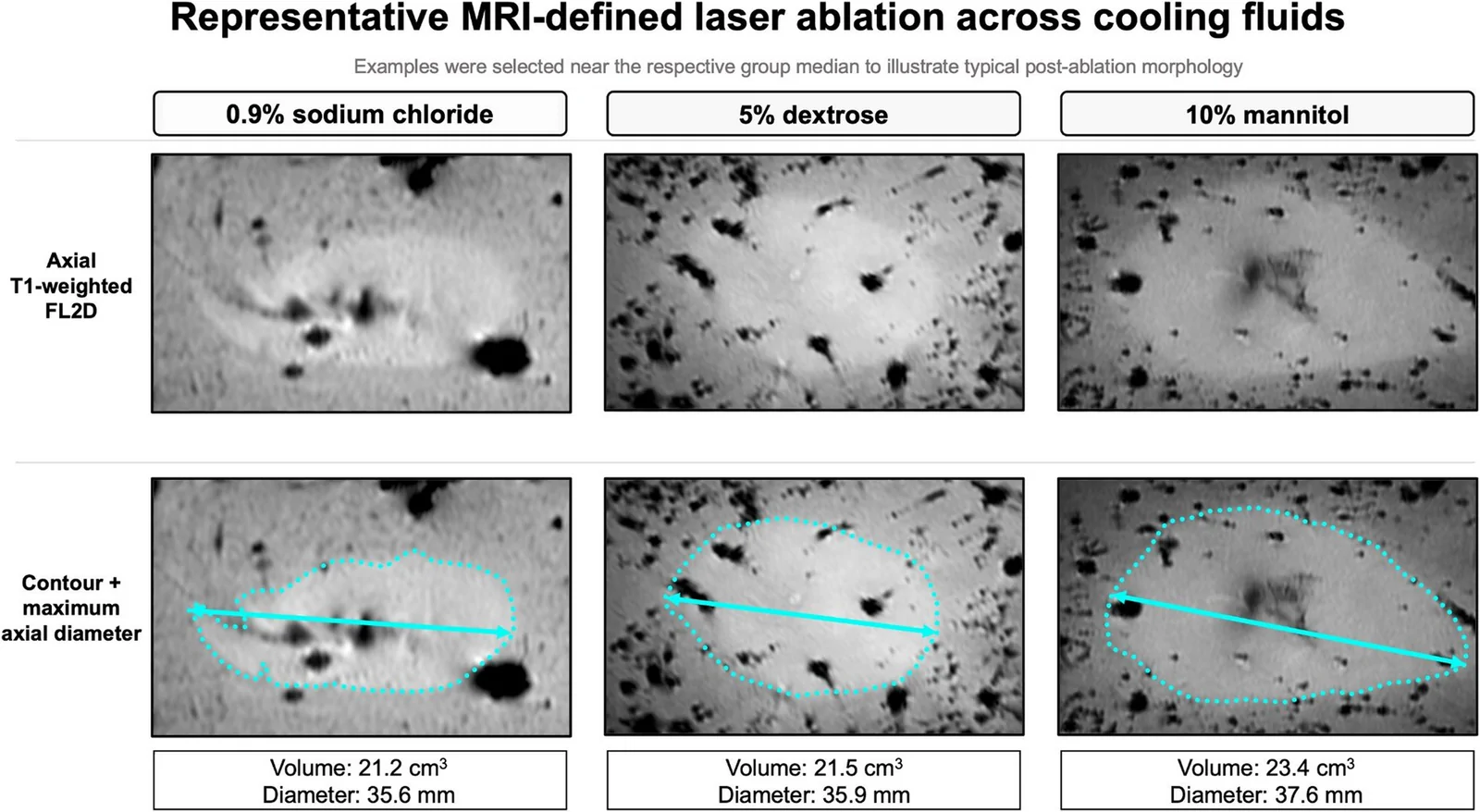
Representative MRI-defined laser ablation zones across cooling fluids. Representative axial T1-weighted FL2D images show post-ablation morphology after laser ablation with internal applicator cooling by 0.9% sodium chloride (NaCl), 5% dextrose in water, and 10% mannitol. The upper row shows the source images without overlay. The lower row shows the same images with dotted contour overlays indicating the manually segmented ablation zone used for volumetry and arrows indicating the maximum axial diameter. Values below each column show the corresponding manual ablation volume in cm³ and maximum axial diameter in mm. Examples were selected near the respective group median to illustrate typical post-ablation morphology rather than extreme findings

This study compared MRI-defined ablation volume among three cooling fluids with different ionic characteristics and osmolarity in ex vivo bovine liver tissue. In a highly standardized ex vivo bovine liver model, 10% mannitol was associated with larger MRI-defined ablation volumes and maximum diameters compared with both 0.9% sodium chloride and 5% dextrose in water, whereas no significant differences were observed between the latter two solutions (Fig. 5). These findings were supported by high agreement between manual and semi-automatic volumetry and excellent intra-rater repeatability, strengthening confidence in the observed group pattern.

Importantly, the experimental protocol reduced procedural variability by standardizing major technical determinants of ablation volume, including laser power, treatment duration, delivered energy, applicator system, cooling flow rate, pre-ablation tissue temperature, MRI acquisition, and volumetric assessment. Within this constrained system, the observed differences are consistent with a fluid-dependent effect at the applicator-tissue interface, although the underlying mechanism cannot be determined from the present data. The observed group pattern does not support a simple distinction between ionic and non-ionic cooling solutions: despite fundamentally different ionic characteristics, 0.9% sodium chloride and 5% dextrose in water produced near-identical ablation volumes. In contrast, 10% mannitol, although also non-ionic, was associated with larger ablation volume. These findings indicate that ionic status alone does not account for the observed group differences but do not identify which fluid property or combination of properties is responsible.

The present study was not designed to determine the physicochemical mechanism underlying these differences. Several fluid-related properties may act in combination at the applicator-tissue interface [18, 19, 21]. However, thermal conductivity, viscosity, vaporization dynamics, interface temperature, effective osmolarity under ablation conditions, and other potentially relevant properties were not directly measured. The higher nominal osmolarity of 10% mannitol should therefore be regarded as a descriptive fluid characteristic rather than evidence of an osmolarity-mediated effect. Whether one or several of these properties contribute to differences in ablation formation remains hypothesis-generating and requires direct assessment in dedicated mechanistic studies.

Internally cooled thermal ablation systems have been shown to reduce carbonization and increase coagulation volume compared with uncooled approaches [21, 22]. In hepatic LA, previous experimental work has focused mainly on MR guidance, applicator configuration, power settings, thermal modelling, and technical optimization [9, 23–25]. A prior ex vivo bovine study compared 0.9% sodium chloride, distilled water, and a 50:50 mixture as cooling fluids and found larger MRI-defined ablation volumes with the mixed solution, while saline and distilled water showed similar volumes despite marked differences in ionic composition [19]. The present study extends that comparison to three clinically available and commonly utilised infusion fluids and reproduces the absence of a significant volume difference between 0.9% sodium chloride and a non-ionic comparator. The larger MRI-defined ablation volumes observed with 10% mannitol are consistent with prior evidence that coolant composition is associated with LA volume, while the repeated similarity between saline and non-ionic comparator fluids argues against electrolyte content alone as a sufficient explanatory variable [26, 27].

Evidence from RFA and MWA also demonstrates that fluid-related factors can influence ablation formation [26, 28, 29]. These findings cannot, however, be directly extrapolated to LA because energy deposition and subsequent tissue heating differ between modalities. In RFA, electrical conductivity directly affects current distribution and resistive heating, whereas MWA generates heat through oscillation of polar molecules within an electromagnetic field. By contrast, LA relies on optical energy absorption followed by thermal conduction within the surrounding tissue. Cooling-fluid effects observed in RFA or MWA may therefore reflect mechanisms that are not directly applicable to LA. Cooling-fluid selection may nevertheless represent a potentially modifiable technical parameter in hepatic LA that warrants further investigation.

The absolute magnitude of the observed effect was modest and was obtained in non-perfused ex vivo tissue. A difference of approximately 2 mm in maximum axial diameter could theoretically become relevant when the achieved ablation zone is close to the intended treatment margin, particularly in situations where margin adequacy is limited by adjacent critical structures. However, the present study does not establish whether a difference of this magnitude translates into improved ablative margin adequacy or local tumor control. The findings should therefore be interpreted as a technical effect observed under standardized experimental conditions rather than evidence of clinical superiority of one cooling fluid.

Our study has limitations which define the scope of interpretation. The ex vivo bovine liver design allowed standardized comparison of the three cooling fluids, but non-perfused tissue does not reproduce physiologic heat-sink effects or post-ablation live tissue response [4, 6, 17, 30, 31]. In clinical liver ablation, perfusion-related heat dissipation can modify local temperature distribution and ablation extent, particularly in proximity to hepatic vessels [4]. Both the absolute ablation volumes and the relative differences observed between cooling fluids may therefore differ under perfused conditions, limiting direct translation of the present findings to clinical practice. Perfused or in vivo liver models would allow assessment of cooling-fluid behaviour under physiologic heat-sink conditions and more realistic ablation growth. The study detected the larger mannitol-associated differences observed in this dataset, while smaller differences between 0.9% sodium chloride and 5% dextrose in water may have remained below the level of detection. Although 33 ablations were evaluable, these originated from only five independent bovine livers. The mixed-effects model accounts for clustering of repeated ablations within the same liver but does not overcome the limited number of independent biological specimens. Between-liver biological variability could therefore only be characterized to a limited extent, and the generalizability of the findings remains restricted. Interface temperature, thermal conductivity, viscosity, and effective osmolarity under ablation conditions were not measured in the present study. No causal contribution of individual fluid properties can therefore be inferred from the observed group differences. Carbonization was likewise assessed only qualitatively and was not prospectively graded using a standardized macroscopic or histological score. The present data consequently do not allow assessment of whether cooling-fluid composition was associated with the extent of carbonization. Dedicated mechanistic studies incorporating direct interface measurements and standardized macroscopic or histological assessment of carbonization would allow these questions to be addressed more specifically. MRI-defined ablation volume represents an imaging endpoint and was not validated against gross pathology or histology in the present study. The measured postablation signal abnormality therefore cannot be assumed to correspond directly to the extent of irreversible coagulation necrosis. Immediate postprocedural MRI at 7T provided high spatial resolution and clear ablation conspicuity, but measurements obtained under these imaging conditions may not be directly interchangeable with volumes derived from standard clinical field strengths and protocols [32]. Gross pathological or histological correlation would be required to determine how accurately 7T MRI-defined ablation volumes represent the extent of coagulation necrosis. Repeatability was assessed within one reader using a four-week washout protocol, whereas inter-rater reproducibility of the quantitative measurements was not assessed. Independent volumetric and diameter measurements by multiple readers would provide additional validation of the imaging methodology. Applicator placement was performed manually without image guidance and may therefore have introduced variability in insertion depth, trajectory, and the local relationship between the applicator and surrounding tissue structures, which can influence heat distribution and ablation geometry [33, 34]. The same placement procedure was used across all groups, and the prespecified repeating 1:1:1 allocation sequence interleaved the three cooling-fluid conditions throughout the experimental series, reducing the likelihood of systematic procedural differences between groups. However, residual placement-related or site-related variability cannot be excluded.

In standardized ex vivo bovine liver, internal applicator cooling with 10% mannitol was associated with larger MRI-defined LA volumes than either 0.9% sodium chloride or 5% dextrose in water, whereas saline and dextrose did not differ significantly. These findings should be considered hypothesis-generating and do not establish clinical superiority of mannitol. Cooling-fluid composition should be standardized and reported in experimental ablation studies. Perfused or in vivo experiments with direct interface measurements and pathological validation are required to determine whether the observed association persists under physiologic conditions and has clinical relevance. 

## Data Availability

The experimental data, MRI-derived volumetric measurements, and statistical analysis data generated and analyzed during the current study are available from the corresponding author on reasonable request. Raw MRI datasets may be provided where technically feasible and subject to institutional data-sharing requirements.

## Declarations

### Clinical Trial

Not applicable. This study was an ex vivo experimental study using postmortem bovine liver tissue and was not a clinical trial.

## Abbreviations

ARRIVE: Animal Research: Reporting of In Vivo Experiments
CI: confidence interval
DICOM: Digital Imaging and Communications in Medicine
FL2D: two-dimensional fast low-angle shot
FL3D: three-dimensional fast low-angle shot
ICC: intraclass correlation coefficient
IQR: interquartile range
LA: laser ablation
MRI: magnetic resonance imaging
NaCl: sodium chloride
Nd:YAG: neodymium-doped yttrium aluminium garnet
RFA: radiofrequency ablation
ROI: region of interest
RRID: Research Resource Identifier
SD: standard deviation
T1w: T1-weighted
T2w: T2-weighted
TSE: turbo spin echo
VIBE: volumetric interpolated breath-hold examination
